# Estrogen promotes the onset and development of idiopathic scoliosis via disproportionate endochondral ossification of the anterior and posterior column in a bipedal rat model

**DOI:** 10.1038/s12276-018-0161-7

**Published:** 2018-11-07

**Authors:** Shuhui Zheng, Hang Zhou, Bo Gao, Yongyong Li, Zhiheng Liao, Taifeng Zhou, Chengjie Lian, Zizhao Wu, Deying Su, Tingting Wang, Peiqiang Su, Caixia Xu

**Affiliations:** 1 Research Center for Translational Medicine, The First Affiliated Hospital of Sun Yat-sen University, Guangzhou, China; 2 Department of Orthopedics, The First Affiliated Hospital of Sun Yat-sen University, Guangzhou, China; 3 Department of Orthopaedics, Sun Yat-sen Memorial Hospital of Sun Yat-sen University, Guangzhou, China

## Abstract

This study aimed to verify the effects of estrogen on the onset and development of adolescent idiopathic scoliosis and the mechanisms associated with these effects by constructing a pubescent bipedal rat model. Experiments were conducted to investigate whether scoliosis progression was prevented by a Triptorelin treatment. One hundred twenty bipedal rats were divided into female, OVX (ovariectomy), OVX + E2, Triptorelin, sham, and male groups. According to a spinal radiographic analysis, the scoliosis rates and curve severity of the female and OVX + E2 groups were higher than those in the OVX, Triptorelin, and male groups. The measurements obtained from the sagittal plane of thoracic vertebrae CT confirmed a relatively slower growth of the anterior elements and a faster growth of the posterior elements between T11 and T13 in the female and OVX + E2 groups than in the OVX and Triptorelin groups. Histomorphometry and immunohistochemistry revealed a significantly longer hypertrophic zone of the vertebral cartilage growth plates that expressed more type X collagen and less type II collagen in the OVX and Triptorelin groups than in the female and OVX + E2 groups. Ki67 immunostaining confirmed an increase in the proliferation of vertebral growth plate chondrocytes in the OVX group compared with the female and OVX + E2 groups. In conclusion, estrogen obviously increased the incidence of scoliosis and curve severity in pubescent bipedal rats. The underlying mechanism may be a loss of coupling of the endochondral ossification between the anterior and posterior columns. Triptorelin decreased the incidence of scoliosis and curve magnitudes in bipedal female rats.

## Orthopedics: Staying ahead of the curve

Treatments that reduce estrogen levels may prevent the development of spinal curvature associated with adolescent idiopathic scoliosis, curvature of the spine. Estrogen has been linked to scoliosis in the past, but the nature of this relationship remains controversial. Researchers led by Caixia Xu and Peiqiang Su of the First Affiliated Hospital of Sun Yat-sen University in Guangzhou, China, used a rat scoliosis model to assess how the absence of estrogen influences the onset and severity of this disorder. They demonstrate that rats with greatly reduced estrogen levels (resulting from ovary removal) have a lower incidence of scoliosis than untreated females, with affected animals exhibiting milder spinal curvature.The researchers observed similar lower incidence in female rats treated with triptorelin, a drug that dramatically reduces estrogen levels, further supporting a prominent role for this hormone in spinal development.

## Introduction

Adolescent idiopathic scoliosis (AIS) is one of the most frequent forms of spinal deformation^1,2^. AIS primarily occurs in pubescent girls^3^. However, the pathogenesis of AIS remains obscure. A number of factors contributing to the pathogenesis of idiopathic scoliosis have been proposed, such as genetic^4,5^, biochemical^6,7^, skeletal^8,9^, and hormonal factors, as well as neuromuscular abnormalities^10–12^.

Various hormones, particularly estrogen, have been reported to play roles in the onset and development of AIS^13,14^. Many abnormal changes in estrogen and estrogen receptors have been observed in most patients with AIS, including serum estrogen concentrations, the cellular response to estrogen, age at menarche, and gene polymorphisms in the estrogen receptor genes, which are closely associated with incidence of scoliosis and curve severity^14^. Nevertheless, the exact role of estrogen in AIS and its mechanisms are controversial. For example, the treatment of *Xenopus laevis* blastulae with a pharmacological dose of 17β estradiol (E2) leads to a notably higher incidence of scoliosis^15^. However, Kulis et al. reported lower serum estrogen concentrations in girls with scoliosis than in the control group^16^. According to Leboeuf, estrogens are not causative factors in AIS, but they likely impact scoliosis by interacting with factors that modulate bone growth, biomechanics, and structure^14^. Therefore, the exact roles and mechanisms by which estrogen participates in the onset and progression of AIS remain unclear, and further research is needed.

Because the quadrupedal rodent spine is unlike the human spine and is not subjected to the same postural and dynamic mechanical forces required for the development of scoliosis^17^, bipedal rat and mouse models have been used to study the mechanism of AIS^18,19^. Therefore, in this study, an adolescent bipedal rat model was chosen to study the roles of estrogen in the onset and development of AIS.

As shown in previous studies, disproportionate growth of the anterior and posterior columns may contribute to the development of AIS^20^. The pathological mechanism that causes the disproportionate growth of the anterior and posterior columns remains unclear. The anterior column includes the vertebral bodies, and the posterior column consists of the pedicles, laminae, superior and inferior articular processes, transverse processes, and spinous processes. The longitudinal growth of the anterior column primarily depends on the growth plates, which grow in the form of endochondral ossification and persist until the girl is aged between 16 and 18 years old^21^. However, endochondral ossification of the posterior column only continues until the girl is 10 years old^22^. We hypothesize that the disproportionate growth of the anterior and posterior columns in patients with AIS is due to a loss of the coupling of endochondral ossification between the anterior and posterior columns during adolescence, the period when the body starts to secrete estrogen.

Therefore, the goal of this study is to investigate the exact roles of estrogen in the onset and development of AIS, and the mechanisms associated with these effects by constructing a pubescent bipedal rat model. Triptorelin, a synthetic decapeptide analog of the natural gonadotropin-releasing hormone (GnRH), reduces estrogen levels in the blood^23^. The ability of a Triptorelin treatment to prevent scoliosis progression in the bipedal female rat model was also investigated.

## Materials and methods

### Experimental animals

One-hundred twenty Sprague–Dawley rats weighing 180–200 g were obtained from the Laboratory Animal Center of Sun Yat-Sen University. All experimental procedures were performed in accordance with the Guidelines of Animal Experiments from the Committee of Medical Ethics, National Health Department of China. In our study, the 120 rats were randomly divided into six groups of 20 animals each: bipedal female rat group (female group), ovariectomized bipedal female rat group (OVX group), ovariectomized plus E2 bipedal female rat group (OVX + E2 group), bipedal female rat plus Triptorelin group (Triptorelin group), sham operation group (sham group), and bipedal male rat group (male group).

The body starts to secrete estrogen at puberty. The circulating serum estrogen levels were measured in the rats aged from 1 to 10 weeks to characterize the exact time course of estrogen secretion in the rats used in the present study (Fig SI). The circulating serum estrogen levels in rats increased from week 1 to week 7, but after week 7, a decreasing trend was observed. The rats were not weaned before week 4, and their endurance was so poor that they were highly likely to die after surgery. Therefore, we selected 5-week-old rats for the operation. At 5 weeks of age, all rats were rendered bipedal by amputating the forelimbs at a high humeral level and the tails at the root under anesthesia, as previously described^18,19^. After removing the forelimbs and tail, the bipedal rats were housed in special high cages with raised food and water to ensure that they maintained a standing posture most of the time; both food and water were gradually elevated in the cage as the bipedal rats grew.

## Evaluation of circulating serum estrogen levels

Blood was collected from the six groups of rats via the orbital vein at 15^th^ week after surgery, and circulating serum estrogen levels were evaluated using the Estrogen (E) ELISA kit for rats (JL12534-96T, Jianglai Biological Science and Technology, China).

## Measurement of scoliosis

Anteroposterior and lateral views of spinal radiographs were captured to evaluate the spinal alignment in all groups 15 weeks after surgery. Scoliosis was measured using Cobb’s method. Using the Cobb’s angle, all bipedal rats were assigned a severity score as follows: 0 = Cobb’s angle of 0° (normal control); 1 = 10–20°(slight deformity); 2 = 21–40°(moderate deformity); and 3 = 41° or higher (severe deformity).

## Measurements of the sagittal plane of the thoracic vertebrae(T8–T13)

Micro-CT was taken on anesthetized bipedal rats at 15^th^ week after surgery. The anterior and posterior height of the vertebral body (VBHa and VBHp, respectively) were measured along these lines. The height of the pedicles (PH) was measured bilaterally, and the maximum value of each pedicle was recorded. Simultaneously, the distance from the inferior edge to the superior edge of the lower pedicle (IPH) was measured. Means and standard deviations (SD) were calculated for the measurements of each vertebra.

## Histology and immunohistochemistry

At 15^th^ week after surgery, the apical vertebrae of the rats in the six groups were immediately fixed with 4% paraformaldehyde for 1 day, followed by decalcification in 10% methylene diamine tetraacetic acid for 3 weeks. The 4 μm-thick paraffin sections were deparaffinized with xylene, rehydrated with decreasing concentrations of ethanol, and finally washed with PBS. Then, sections were stained with hematoxylin and eosin (HE) (Sigma-Aldrich, St. Louis, MO, USA) to examine the cartilage structure. For immunohistochemistry, rehydrated sections were sequentially incubated with a pepsin solution at 37 °C for 10 min and 3% H_2_O_2_ for 10 min. Sections were blocked with 1/100 diluted goat serum in TBS/sapon in for 15 min, and then incubated overnight at 4 °C with rabbit anti-rat type II collagen polyclonal antibodies (Abcam, Cambridge, UK) and type X collagen polyclonal antibodies (Abcam, Cambridge, UK), diluted at 1:1000. Afterwards, biotinylated goat anti-rabbit IgG (EarthOx, San Francisco, USA) was applied for 30 min. Sections were incubated with a peroxide-conjugated streptavidin working solution and stained with 3,3′-diaminobenzidine tetrahydrochloride (DAB) (Jinshan Jinqiao, Beijing, China). Staining was visualized using an Olympus IX71 microscope. The Ki67 protein was detected by immunofluorescence staining. Sections were incubated with an anti-Ki67 primary antibody (1:150, Abcam, Cambridge, UK) overnight at 4 °C, followed by a 60-min incubation with an Alexa Fluor 555-labeled secondary antibody (Cell Signaling Technology, USA) at room temperature. Nuclei were labeled with DAPI (0.5 μg/ml, Cell Signaling Technology, USA). Quantification of the percentage of Ki67-positive cells was conducted using Image J software (version1.51). The percentage of Ki67-positive cells was calculated by dividing the number of Ki67-positive cells by the number of cell nuclei.

## Statistical analysis

All analyses were performed with statistics software (SPSS11), and *p* < 0.05 was considered statistically significant. The chi-square test (*χ*^2^ test) was used to compare the incidence of scoliosis and the percentages of Ki67-labeled cells among and within the groups. Analysis of variance (ANOVA) was used to compare the circulating serum estrogen levels and the mean heights of the anterior and posterior columns of the vertebral body among and within groups. Subgroup analyses were performed using Tukey’s HSD test.

## Results

### Circulating serum estrogen levels in the bipedal rat model

**Fig. 1:**
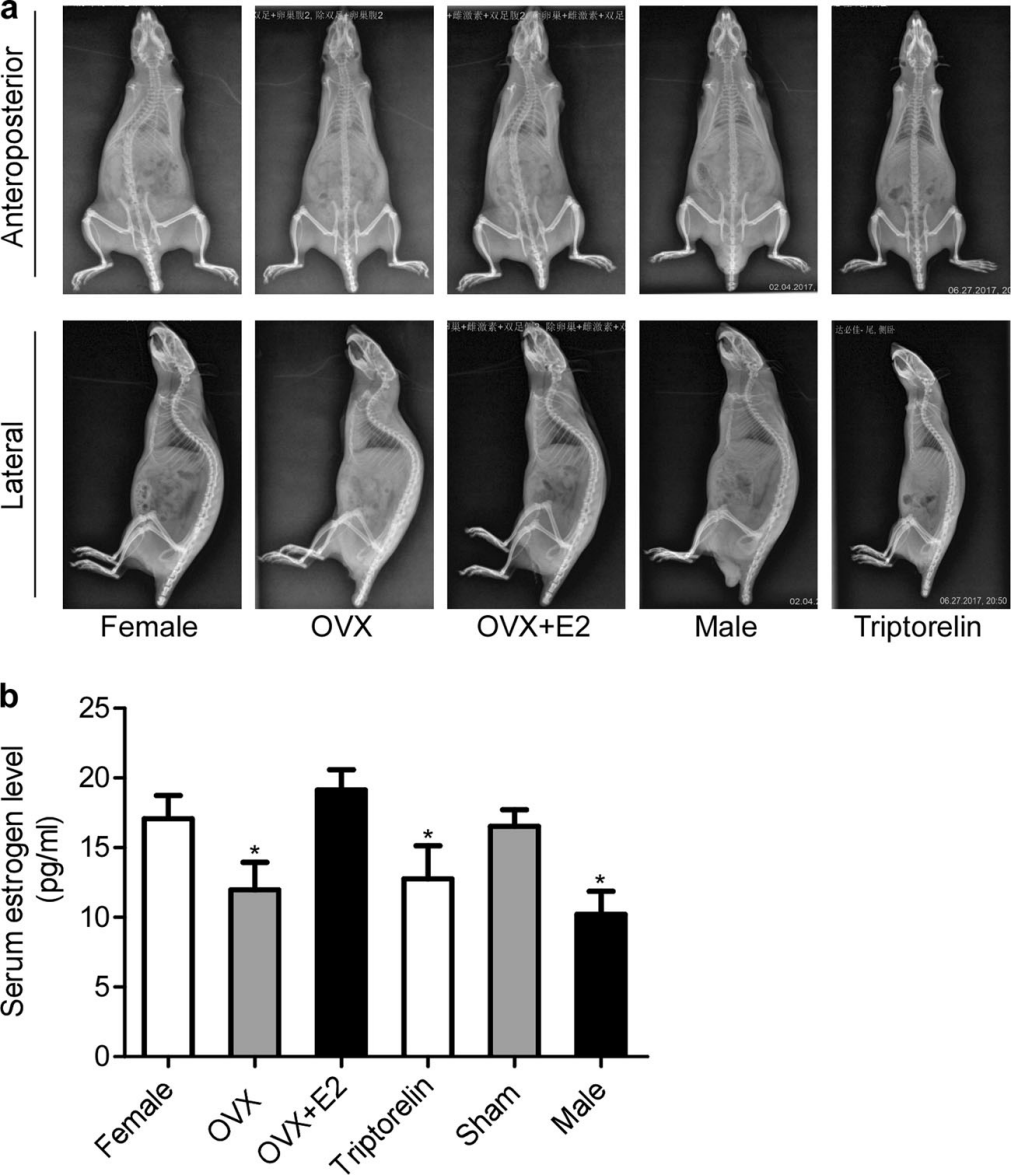
The relationship between the onset of scoliosis and serum estrogen levels in bipedal rats. **a** At 15^th^ week after surgery, an obvious curve was observed on the radiographs from the female group and OVX + E2 group compared to the OVX, Triptorelin, and male groups. **b** Serum estrogen levels in all groups. At 15^th^ week after surgery, significantly lower circulating serum estrogen levels were observed in the OVX group, Triptorelin group, and male group than in the female group (*p* < 0.05); *p < 0.05 compared with the female group

Circulating serum estrogen levels in all groups were evaluated at 15^th^ week after surgery to investigate the association between estrogen levels and scoliosis (Fig. 1b). The serum estrogen concentration in the female group (17.08 ± 1.65 pg/ml) fluctuated within the normal range. Significantly lower circulating serum estrogen levels were observed in the OVX group (11.97 ± 1.97 pg/ml) than in the female group (*p* < 0.05). The serum estrogen concentration exceeded 15 pg/ml in the female group(17.08 ± 1.65 pg/ml) and the OVX + E2 group (19.14 ± 1.45 pg/ml), but the statistical analysis did not show a significant difference between these two groups (*p* > 0.05). The serum estrogen level in the Triptorelin group (12.77 ± 2.36 pg/ml) was reduced to the castration level, and the statistical analysis also showed a significant difference between the Triptorelin group and female group (*p* < 0.05). Figure 1 shows the serum estrogen levels in all groups at 15^th^ week after surgery.

### Radiology

Anteroposterior and lateral views of spinal radiographs are shown in Fig. 1a, and overall scoliosis rates recorded at 15^th^ week after surgery are shown in Table 1. The incidence of scoliosis was 85% in the female group, 25% in the OVX group, 60% in the OVX + E2 group, 30% in the Triptorelin group, 75% in the sham group, and 20% in the male group. Noticeably higher scoliosis rates were observed in the female group and OVX + E2 group (85% and 60%, respectively) than in the OVX group (25%, *p* < 0.05). Moreover, the Triptorelin group showed lower scoliosis rates (30%) than the female group (*p* < 0.05).

**Table 1:** The incidence of scoliosis and scoliosis severity in all groups of bipedal rats

Group	Total, *n*	Animals with scoliosis	Animals without scoliosis	Incidence of scoliosis	Slight (10–20 °)	Moderate (20–40°)	Severe deformity (>40°)
Female group	20	17	3	85%*	10 (50%)	6 (30%)	1 (5%)
OVX group	20	5	15	25%**	2 (10%)	3 (15%)	0
OVX + E2 group	20	12	8	60%*	8 (40%)	3 (15%)	0
Triptorelin group	20	6	15	30%**	5 (25%)	0	0
Male rat group	20	4	16	20%**	4 (20%)	0	0
Sham group	20	15	0	75%*	15 (75%)	0	0

*Significantly different from the OVX group (*p* < 0.05)

**Significantly different from the female group (*p* < 0.05)

This table shows the incidence of scoliosis and scoliosis severity in the four groups at 15^th^ week after surgery. The incidences of scoliosis in the female and OVX + E2 groups were significantly higher than the OVX group (*p* < 0.05), and the incidences of scoliosis in the Triptorelin group and male group were significantly lower than the female group (*p* < 0.05). The incidence of slight deformity, moderate deformity, and severe deformity was 50%, 30%, and 5%, respectively, in female group, and 40%, 15%, and 0%, respectively, in the OVX + E2 group. However, the incidences of slight and moderate deformities in the OVX group (10% and 15%, respectively) and Triptorelin group (25% and 0%, respectively) were less than the female group and OVX + E2 group, and no rats in these two groups presented with a severe deformity. **p < 0.05 compared with the female group, *p < 0.05 compared with the OVX group

Scoliosis was measured using Cobb’s method. Using the Cobb’s angle, all bipedal rats were assigned a severity score as follows: 0 = Cobb’s angle of 0° (normal control); 1 = 10–20° (slight deformity); 2 = 21–40° (moderate deformity); and 3 = 41° or higher (severe deformity). The incidences of slight, moderate, and severe deformities were 50%, 30%, and 5%, respectively, in the female group, and 40%, 15%, and 0%, respectively, in the OVX + E2 group. However, the incidences of slight and moderate deformities in the OVX group (10% and 15%, respectively) and the Triptorelin group (25% and 0%, respectively) were less than the female group and the OVX + E2 group, and no rats in these two groups presented with a severe deformity. Based on these findings, estrogen not only increases the incidence of scoliosis but also increases the rate of the progression of the deformity in bipedal rats.

### Measurements of the sagittal plane of the thoracic vertebrae (T8–T13)

**Fig. 2:**
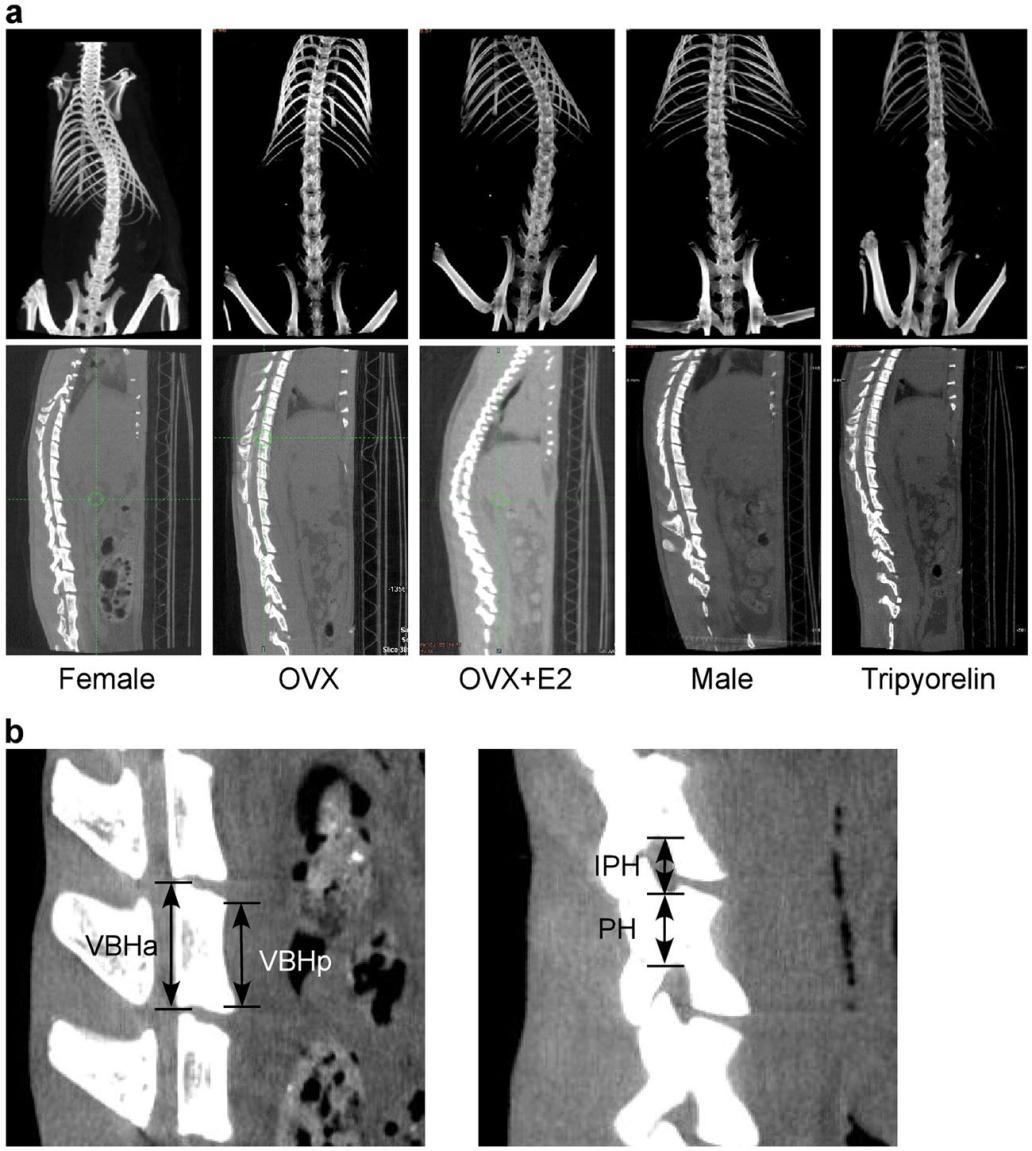
The spine CT scans of the four groups. **a** Coronal CT scans of the four groups recorded at 15^th^ week after surgery. **b** Sagittal CT scan showing how the measurements of the vertebral body (VBHa and VBHp) and the pedicle (PH and IPH) were obtained

Spine CT images of all groups were captured to investigate the abnormal differential growth of the anterior and posterior elements of the thoracic vertebrae in bipedal rats with scoliosis (Fig. 2a). The height of the vertebral bodies, the pedicular height (PH), and the interpedicular distance (IPH) were measured as shown in Fig. 2b and Table 2. Between T11 and T13, the vertebral bodies (both VBHa and VBHp) were consistently shorter in the female group and OVX + E2 group than in the OVX group (*p* < 0.05). However, in the posterior column, the PH and the IPH were increased in the female group and the OVX + E2 group. Both VBHa and VBHp were longer in the Triptorelin group than in the female group, while the PH and IPH were shorter. The differential growth along the longitudinal axis between the posterior and anterior elements of each thoracic vertebra in the female group and OVX + E2 group was significantly greater than the OVX group (*p* < 0.05).

**Table 2:** Detailed sagittal measurements of the thoracic vertebrae (T8–T13) in the four groups

	T13	T12	T11	T10	T9	T8
*VBHa (mm)*						
Female group	4.02 ± 0.15*	3.87 ± 0.08*	4.03 ± 0.15*	4.07 ± 0.10*	3.96 ± 0.14*	3.95 ± 0.12
OVX group	4.37 ± 0.18**	4.29 ± 0.28**	3.79 ± 0.16**	3.84 ± 0.20**	3.80 ± 0.16**	4.04 ± 0.13
OVX + E2 group	4.27 ± 0.43	3.86 ± 0.41	4.04 ± 0.22	4.15 ± 0.22*	3.92 ± 0.18	4.11 ± 0.18
Triptorelin group	4.52 ± 0.13	3.92 ± 0.20	3.72 ± 0.18**	3.78 ± 0.13**	3.79 ± 0.07	3.71 ± 0.18**
*VBHp (mm)*						
Female group	4.91 ± 0.19*	4.77 ± 0.11*	4.34 ± 0.24*	4.27 ± 0.10	4.11 ± 0.10	4.08 ± 0.04*
OVX group	5.34 ± 0.17**	5.07 ± 0.16**	4.62 ± 0.13**	4.31 ± 0.13	4.14 ± 0.08	4.27 ± 0.12**
OVX + E2 group	5.28 ± 0.42	4.73 ± 0.57	4.59 ± 0.17	4.23 ± 0.14	4.12 ± 0.11	4.09 ± 0.11*
Triptorelin group	5.34 ± 0.23**	4.79 ± 0.27	4.51 ± 0.24	3.95 ± 0.10**	4.05 ± 0.11	3.98 ± 0.05
*PH (mm)*						
Female group	4.20 ± 0.17	3.95 ± 0.18*	3.42 ± 0.23*	2.82 ± 0.39*	2.68 ± 0.24*	2.43 ± 0.16*
OVX group	3.88 ± 0.30	3.26 ± 0.37**	2.95 ± 0.29**	2.27 ± 0.41**	2.37 ± 0.12**	2.21 ± 0.08**
OVX + E2 group	4.18 ± 0.39	3.29 ± 0.35	2.83 ± 0.39	2.44 ± 0.17	1.87 ± 0.44*	2.08 ± 0.18
Triptorelin group	4.17 ± 0.31	3.40 ± 0.31**	3.05 ± 0.27**	2.58 ± 0.30	2.44 ± 0.26	2.18 ± 0.16
*IPH (mm)*						
Female group	1.64 ± 0.04*	1.56 ± 0.11	1.65 ± 0.23	1.61 ± 0.15*	1.50 ± 0.14	1.83 ± 0.19
OVX group	1.38 ± 0.09**	1.45 ± 0.21	1.47 ± 0.24	1.32 ± 0.16**	1.43 ± 0.17	1.88 ± 0.11
OVX + E2 group	1.42 ± 0.16	1.59 ± 0.57	1.50 ± 0.29	1.59 ± 0.35	1.41 ± 0.21	1.86 ± 0.22
Triptorelin group	1.44 ± 0.23	1.39 ± 0.15	1.28 ± 0.22	1.37 ± 0.28	1.51 ± 0.10	1.79 ± 0.22

*Significantly different from the OVX group (*p* < 0.05)

**Significantly different from the female group (*p* < 0.05)

This table shows the detailed sagittal measurements of the thoracic vertebrae (T8–T13) in all groups of bipedal rats. Between T11 and T13, both VBHa and VBHp were consistently shorter in the female and OVX + E2 groups than in the OVX group(*p* < 0.05), while in the posterior column, the pedicular height (PH) and the interpedicular distance (IPH) were increased in the female and OVX + E2 groups compared to the OVX group (*p* < 0.05). Both VBHa and VBHp were longer in the Triptorelin group than in the female group, while the PH and IPH were shorter; an opposite trend was observed in the scoliotic spines from the female and OVX + E2 groups between T8 and T10, with longer vertebral bodies for both VBHa and VBHp in the anterior column and shorter PH and IPH in the posterior column than the OVX group (*p* < 0.05). *p < 0.05 compared with the female group, #*p* < 0.05 compared with the OVX group

An opposite trend was observed in the scoliotic spines between T8 and T10 of the female group and OVX + E2 group, with longer vertebral bodies for both VBHa and VBHp in the anterior column and shorter pedicles and IPH in the posterior column than the OVX group and the Triptorelin group.

### Histomorphometry

**Fig. 3:**
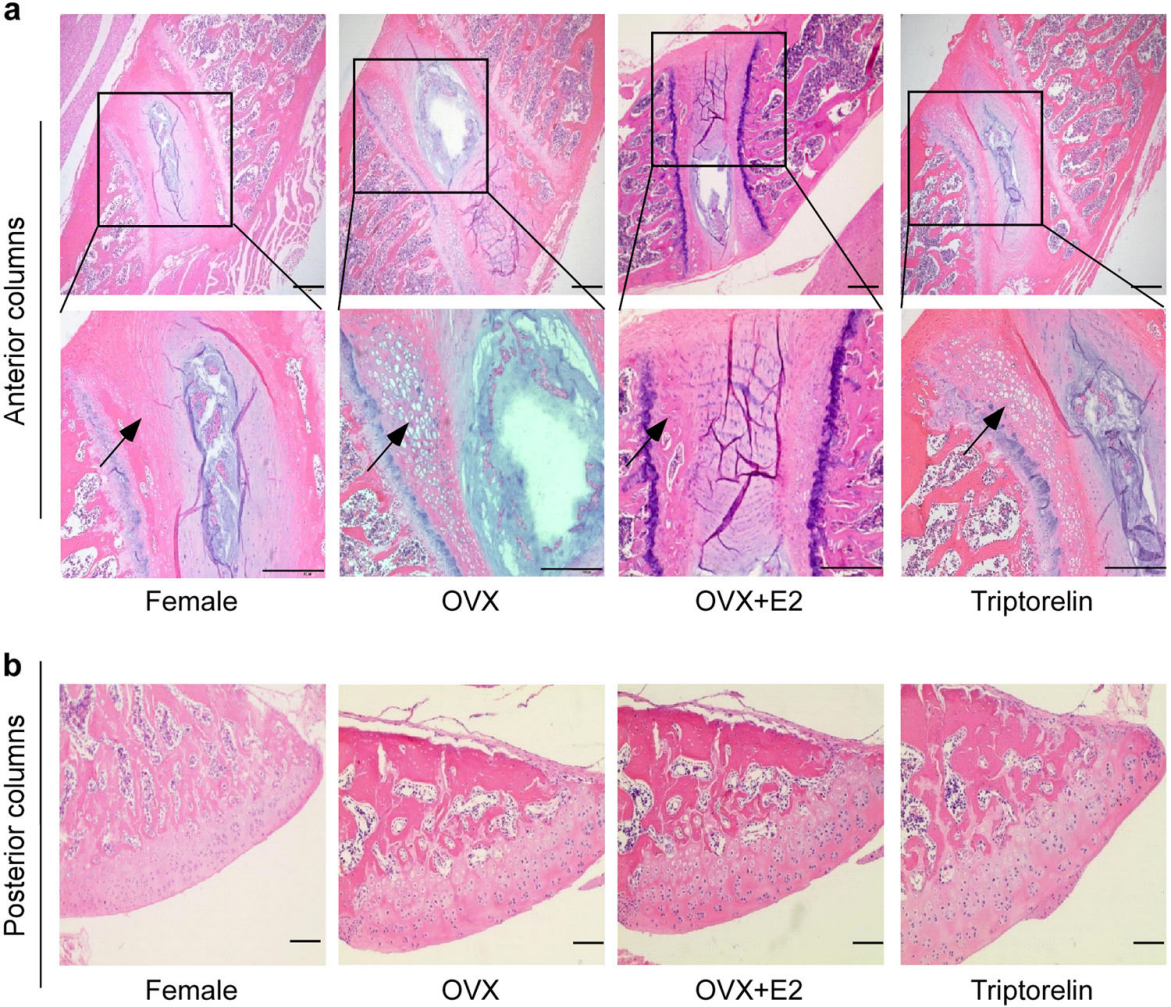
Histomorphometric analysis of the vertebral growth plate cartilage in the four groups. **a** H&E staining of anterior columns of the apical vertebra in the four groups of bipedal rats; the lower panel shows higher magnification images of the data shown in the upper panel (×100 and ×200). The cartilage hypertrophic zones (black arrow) were indicated. The height of the hypertrophic zone in the vertebral growth plate was significantly larger in the OVX and Triptorelin groups than in the female and OVX + E2 groups. The heights of the hypertrophic zone are presented below the images (Table 3). **b** H&E staining of posterior columns of the apical vertebra in the four groups of bipedal rats (×100). The height of the hypertrophic zone in the vertebral growth plate was significantly larger in the OVX and Triptorelin groups than in the female and OVX + E2 groups. No difference was observed in the articular cartilage of posterior columns among the four groups. Scale bar, 100 μm

The chondrocytes in vertebral growth plates were divided into resting, proliferative, hypertrophic, and mineralization zones. Figure 3 and Table 3 show the detailed histomorphometric analysis of the vertebral growth plate cartilage. In anterior columns, the height of the hypertrophic zone in the vertebral growth plate was significantly increased in the OVX group (271.34 ± 45.16 μm) and the Triptorelin group (247.50 ± 45.41 μm) compared to the female group and OVX + E2 group (103.33 ± 15.20 and 118.83 ± 19.60 μm, respectively). A significant difference in the height of the hypertrophic zone was observed between the OVX group and the female group (*p* < 0.05) and between the OVX group and OVX + E2 group (*p* < 0.05).The heights of the hypertrophic zone in the Triptorelin group and OVX group were 247.50 ± 45.41 and 271.34 ± 45.16 μm, respectively, but the statistical analysis did not show any significant difference between these two groups (*p* > 0.05). No differences were observed in the articular cartilage of posterior columns among the four groups.

**Table 3:** Histomorphometry of the hypertrophic zone in the growth plate of the four groups

HZ thickness (μm) or HZ area (×10^5^ μm^2^)	Female group (*n* = 6)	OVX group (*n* = 6)	OVX + E2 group (*n* = 6)	Triptorelin group(*n*=6)
Height of the hypertrophic zone (μm)	103.33 ± 15.20*	271.34 ± 45.16**	118.83 ± 19.60*	247.50 ± 45.41**
Area of the hypertrophic zone (×10^5^ μm^2^)	0.87 ± 0.12*	1.72 ± 0.33**	0.77 ± 0.07*	1.66 ± 0.25**

∗Significantly different from the OVX group (*p* < 0.05)

**Significantly different from the female group (*p* < 0.05)

This table shows the height of the hypertrophic zone in the vertebral growth plate among the four groups. Significantly greater mean heights of the hypertrophic zone were observed in the OVX and Triptorelin groups than in the female and OVX + E2 groups (*p* < 0.05). *p < 0.05 compared with the OVX group, **p < 0.05 compared with the female group. HZ represents the hypertrophic zone

### Immunohistochemistry

**Fig. 4:**
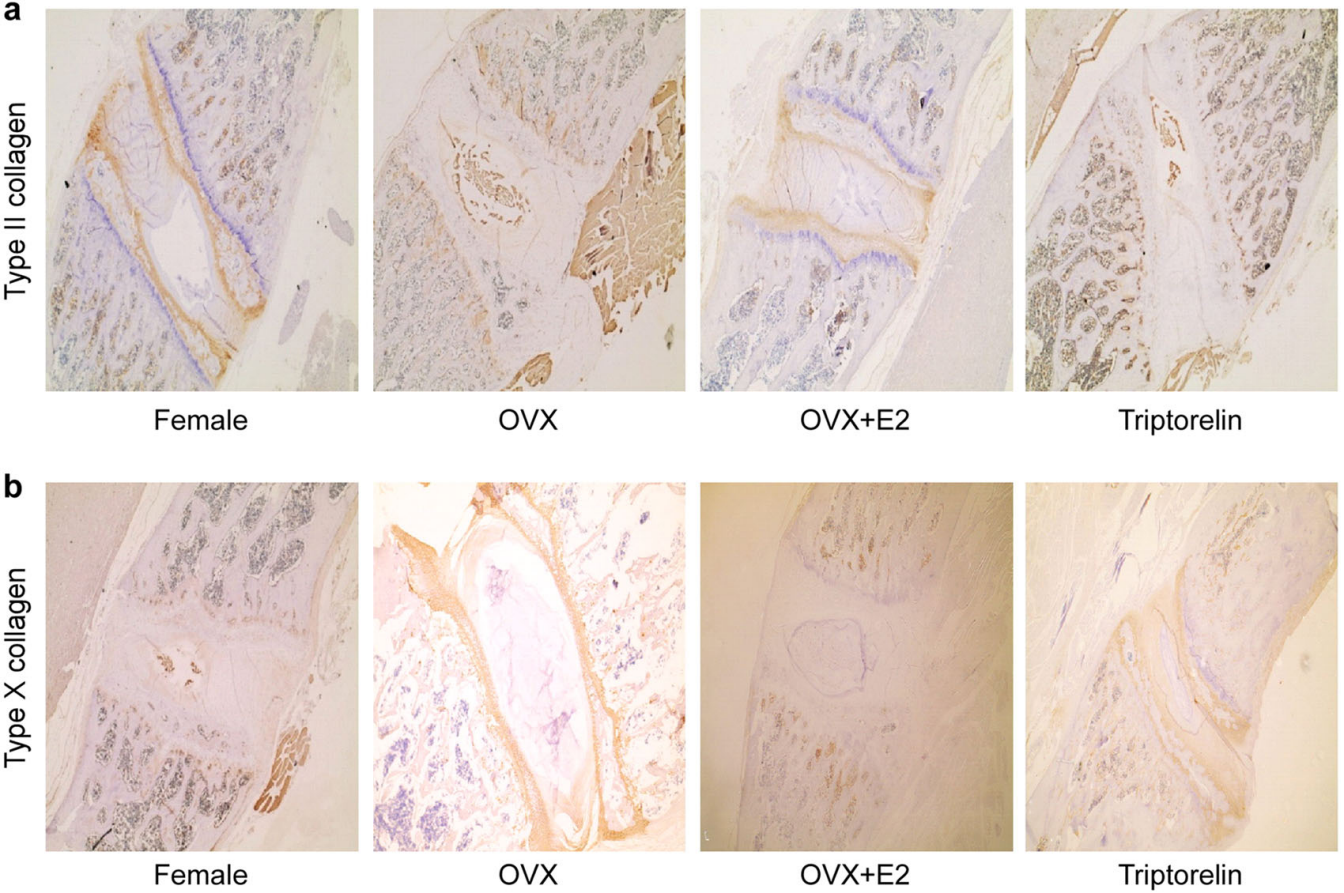
Immunostaining for Col II and Col X in the vertebral growth plate cartilage from the four groups. **a** Cytoplasmic staining of Col II in the photographs showed lower Col II expression in the OVX and Triptorelin groups than in the female and OVX + E2 groups. **b** Cytoplasmic staining of Col X in the photographs revealed increased expression of Col X in the OVX and Triptorelin groups than in the female and OVX + E2 groups

Immunohistochemical staining of the vertebral cartilage growth plates of the apical vertebrae from each group was performed to investigate the differences in Col II and Col X expression among the female, OVX, OVX + E2, and Triptorelin groups. Cytoplasmic staining of Col II and Col X in the photographs demonstrates increased expression of Col X in the OVX and Triptorelin groups compared to the female group and OVX + E2 group, while the OVX and Triptorelin groups showed lower expression of Col II than the female group and OVX + E2 group (Fig. 4).

### Proliferative activity of the vertebral growth plate chondrocytes

**Fig. 5:**
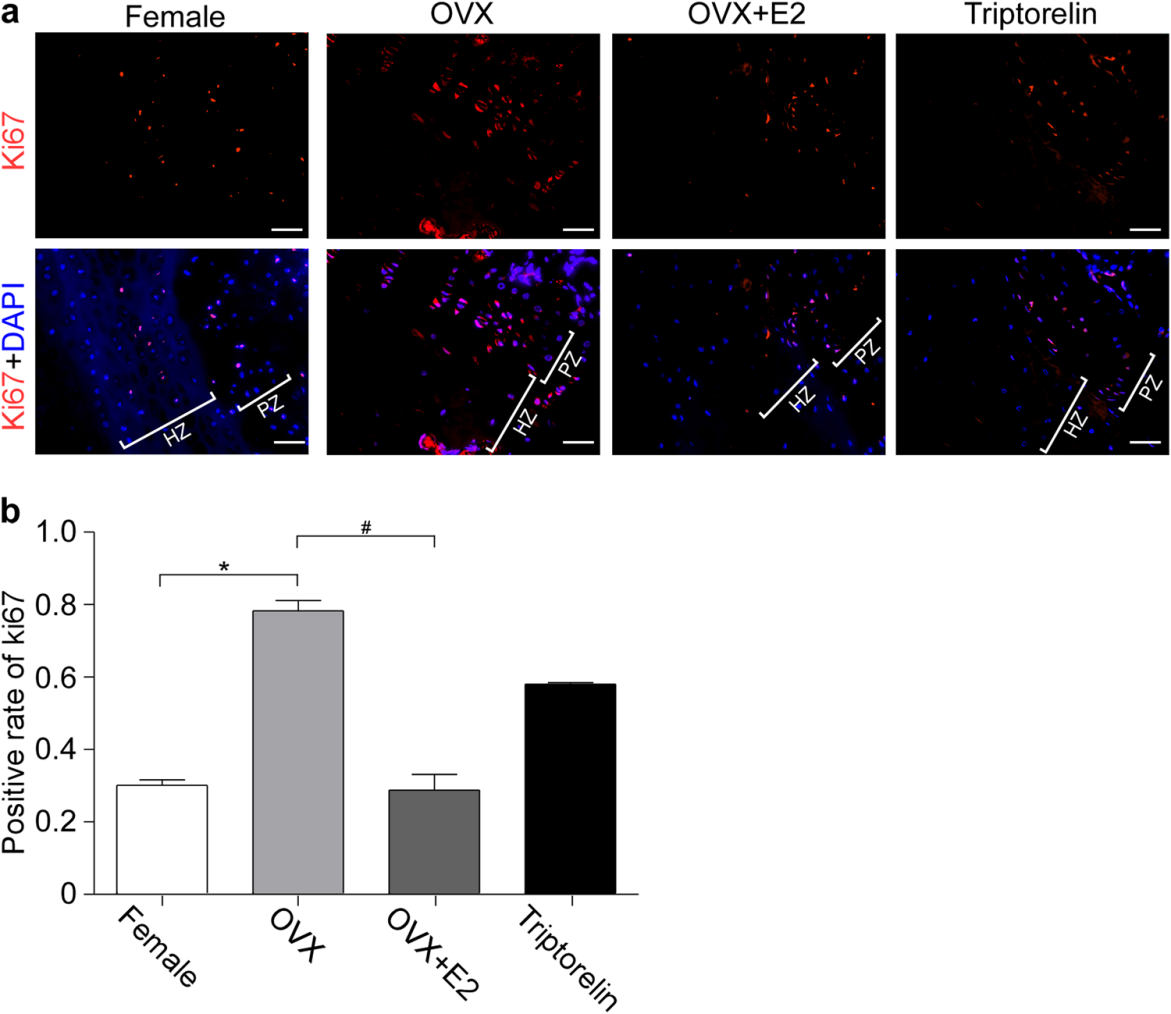
Proliferation of the vertebral growth plate chondrocytes. **a** An anti-Ki67 antibody was used to label proliferating chondrocytes (red fluorescence) and DAPI was used to label the nuclei (blue fluorescence) of the vertebral growth plate chondrocytes. All experiments were repeated three times with consistent results, and representative images are shown. **b** Positive rate of Ki67-labeled nuclei in the images shown in (**a**) was quantified. Scale bars, 25 μm. In (**b**), *means *P* < 0.05 compared with the female group, by the chi-square test (*χ*^2^ test). HZ represents the hypertrophic zone, and PZ represents the proliferative zone

Ki67 immunostaining was performed to label and determine the number of proliferating cells within the vertebral growth plate chondrocytes. The Ki67 protein is a marker of proliferating cells. Chondrocytes displaying intense red staining were defined as Ki67-positive cells and were mainly located in the proliferative zone of the growth plate of the bipedal rats (Fig. 5). The percentage of Ki67-positive cells in the OVX group (78.87%) was obviously increased compared with the female group and OVX + E2 group (30.13% and 28.8%, respectively; *p* < 0.05; Fig. 5). No difference was observed in the percentage of Ki67-positive cells between the female group and the Triptorelin group (30.13% vs. 58.03%, respectively; *p* > 0.05; Fig. 5). Thus, high estradiol levels during late puberty reduced the proliferative activity of chondrocytes in the growth plate.

## Discussion

This study investigated the possible effects of estrogen on the incidence and progression of the scoliotic curves in a bipedal rat model. Estrogen significantly increased the incidence of scoliotic curves and curve severity in bipedal rats at puberty, indicating that estrogen is a factor contributing to AIS. Triptorelin was as effective as ovariectomy in preventing the progression of scoliotic curves.

The association between estrogen levels and scoliosis has been previously reported^24^. According to some studies, estrogen is a factor contributing to the onset of scoliosis, while other studies have claimed that estrogen may be an inhibiting factor for scoliosis^16^. In the present study, we investigated the potential association between spinal deformation and the serum estrogen levels in bipedal rat models. We observed a higher incidence of scoliosis and greater curve severity in the female and OVX + E2 groups than in the OVX and Triptorelin groups. However, higher serum estrogen levels were detected in the female and OVX + E2 groups than in the OVX and Triptorelin groups. Thus, higher circulating estrogen levels obviously increase the risk of scoliosis in bipedal rat models, indicating that estrogen is a factor contributing to AIS. Based on data from some clinical studies, significant differences in serum E2 concentrations are not observed between controls and girls with AIS^25,26^. A possible explanation for the difference in the results obtained between our study and those clinical studies may be that many contributing factors are observed in clinical research, and a compensatory decrease in estrogen levels may occur after the occurrence of scoliosis. However, in the bipedal female rat model, we can maximize the interference effect of estrogen, and thus the impact of estrogen on scoliosis maybe more objectively reflected.

Triptorelin is a synthetic decapeptide analog of natural GnRH. After treatment with triptorelin, estrogen levels in the blood will be reduced to the castration level within 2 weeks. In our study, the Triptorelin treatment reduced the incidence of scoliosis caused by estrogen. Our results on scoliosis rates in bipedal rat models differ from the results obtained by Demirkiran and colleagues^27^. The authors found that treatment with Tamoxifen (TMX) or Raloxifene (RLX) did not reduce the incidence of scoliosis, but TMX or RLX decreased the rate of progression of the deformity. TMX and RLX are both selective estrogen receptor modulators (SERMs), and their positive effects are mediated by a regulatory effect on estrogen or estrogen-regulated proteins through the SERM mechanisms. The study by Demirkiran used melatonin-deficient bipedal mice as the animal model, while our study used bipedal female rats as the animal model. Demirkiran et al. postulated that the underlying mechanism by which RLX decreased curve magnitudes may be the deceleration of the growth rate of the vertebral column and an increase in bone density, causing the early maturation of growth plates.

As shown in previous studies, the disproportionate growth of the anterior and posterior columns may contribute to the development of AIS. As shown in the study by Guo^20^, scoliotic spines have longer vertebral bodies between T1 and T12 in the anterior column and shorter pedicles with a larger IPH in the posterior column than controls. In our study, the measurements from the micro-CT of the spine in the sagittal plane confirmed a relatively slower growth of the anterior elements and a faster growth of the posterior elements of vertebral bodies between T11 and T13 in the female group and OVX + E2 group. Based on these results, estrogen may be correlated with the extent of disproportionate anteroposterior growth. A possible explanation for the difference in the results obtained in our study and of the study by Guo and colleagues^20^ may be the differences in the pressures on the anterior and posterior columns of the spine between humans and rats. The study by Guo included a group of 83 girls with AIS and a group of 22 age-matched controls, while our study used the bipedal female rat model.

Longitudinal growth of the spine is mainly due to the growth of the cartilage area, including the growth plates of the vertebral body and articular cartilage. The longitudinal growth of the anterior column of the spine, including the growth of the vertebral body, is mainly due to the development of the vertebral growth plate by endochondral ossification, while the longitudinal growth of the posterior column is mainly derived from the articular cartilage. In the study by Kronenberg^28^, chondrocyte hypertrophy was a key step in endochondral ossification, and at this stage, chondrocytes exhibited a dramatically altered morphology and size. Hypertrophic chondrocytes generate a mineralizing cartilage matrix and secrete molecules required to induce osteoblastogenesis. The thickness of the hypertrophied cartilage zone, the proliferative activity of chondrocytes, the area of the cell nests, and the number of cells in the nests are the main indicators used to evaluate the development of endochondral ossification^29^. Our study reveals that the hypertrophic zone of the vertebral cartilage growth plates was significantly longer in the OVX and Triptorelin groups than in the female and OVX + E2 groups. Hypertrophic chondrocytes express Col X, which contributes to the degradation of cartilage and replacement by bone. Therefore, Col X is a marker of cartilage hypertrophy. While Col II is expressed by chondrocytes during proliferation, its expression is ultimately terminated when chondrocytes undergo hypertrophy in the growth plate cartilage. In our study, we observed increased expression of Col X in the vertebral cartilage growth plates of the OVX and Triptorelin groups compared to the female and OVX + E2 groups, while decreased expression of Col II was observed in the vertebral cartilage growth plates of the OVX and Triptorelin groups. Chondrocyte hypertrophy is responsible for approximately 60% of skeletal growth^30^. The height of chondrocytes increases approximately 6-to 10-fold during the hypertrophic process, and thus hypertrophic differentiation is an important contributor to longitudinal growth^31^. Chondrocyte hypertrophy may be associated with disc degeneration. The hypertrophic differentiation of endplate chondrocytes has been reported to lead to matrix calcification, and endplate cartilage calcification is the initial step in disc degeneration^32^. We speculate that chondrocyte hypertrophy is largely responsible for skeletal growth during puberty, whereas chondrocyte hypertrophy is sometimes associated with disc degeneration in middle-aged and elderly stages. The rats examined in this study were in late puberty (4 months of age), and chondrocyte hypertrophy is largely responsible for skeletal growth at this stage. In our study, we observed increased proliferation of vertebral growth plate chondrocytes in the OVX group compared with the OVX + E2 and female groups. During early puberty, estrogens enhance skeletal growth, whereas high estradiol levels during late puberty reduce the size of the proliferative zone and the percentage of proliferating chondrocytes in growth plate^33^, consistent with our results. Based on these results, estrogen obviously decreases the development of endochondral ossification in the vertebral cartilage growth plates. No difference in cartilage zones in the posterior columns were observed among the groups. We speculated that the disproportionate growth between the anterior and posterior columns in bipedal rats is due to a loss of the coupling of endochondral ossification between the anterior and posterior columns during adolescence. This hypothesis is consistent with the results from the study by Zhu and colleagues^34^. The authors observed a significant difference in the histological grading and chondrocyte activity of growth plate cartilage between the anterior and posterior column in patients with AIS.

In conclusion, estrogen increases the incidence and curve severity of scoliosis in bipedal female rats. One probable underlying mechanism may be the effects on the imbalance of endochondral ossification between the anterior and posterior columns during adolescence, which causes the disproportionate growth between the anterior and posterior columns. A reduction in the estrogen levels appears to be useful in controlling scoliotic deformity in bipedal female rats, and it may be useful as a potentially new medical treatment for AIS. In the present study, a triptorelin treatment reduced the incidence of scoliosis. These results may provide new insights into the potential targets for the prevention and treatment of adolescent idiopathic scoliosis. Further studies will be required before these findings are translated to humans.

## Electronic supplementary material

Supplementary Materials
