# Supplementary material for: Estrogen promotes the onset and development of idiopathic scoliosis via disproportionate endochondral ossification of the anterior and posterior column in a bipedal rat model
Source: Exp Mol Med. 2018 Nov 7;50(11):144. doi: 10.1038/s12276-018-0161-7 (PMC6220154; doi:10.1038/s12276-018-0161-7)
Supplement: Supplementary file 1 — Supplementary Materials [file 12276_2018_161_MOESM1_ESM.docx]

**SUPPL.FIGURE**


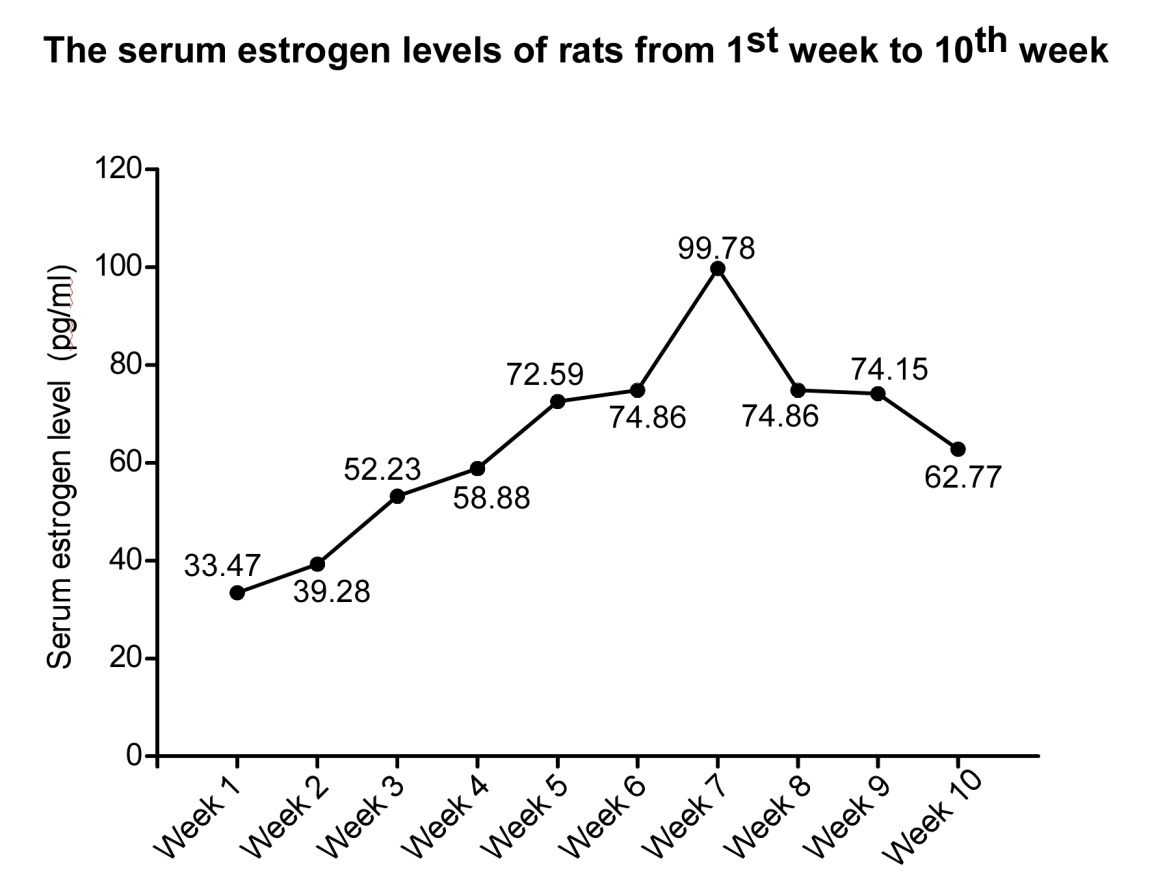


**Fig *Supplementary I.*** The circulating serum estrogen levels from week 1 to week 10 of age were evaluated in the rats used in this study (Fig *SI*). The circulating serum estrogen levels of rats increased from week 1 to week 7, but after week 7, it displayed a downtrend.
